# Comparison of clinical efficacy of single-incision and traditional laparoscopic surgery for colorectal cancer: A meta-analysis of randomized controlled trials and propensity-score matched studies

**DOI:** 10.3389/fonc.2022.997894

**Published:** 2022-10-13

**Authors:** Fang-han Li, De-xin Zeng, Li Chen, Cheng-fei Xu, Ling Tan, Pan Zhang, Jiang-wei Xiao

**Affiliations:** Department of Gastrointestinal Surgery, Clinical Medical College and The First Affiliated Hospital of Chengdu Medical College, Chengdu, China

**Keywords:** single-incision laparoscopic surgery (SILS), conventional laparoscopic surgery (CLS), colorectal cancer, randomized controlled trials, meta-analysis, complication

## Abstract

**Background:**

Single-incision laparoscopy surgery (SILS) is a new laparoscopic technique that has emerged in the past decade. Whether it has advantages over conventionl laparoscopy surgery (CLS) is inconclusive. This article aimed to compare the short- and long-term outcomes of single-incision laparoscopic surgery and conventional laparoscopic surgery for colorectal cancer through high-quality literature text mining and meta-analysis.

**Methods:**

Relevant articles were searched on the PubMed, Embase, and Cochrane Library databases from January 2012 to November 2021. All data was from randomized controlled trials (RCTs) in order to increase the confidence of the analytical results.The main outcomes were intraoperative and postoperative complications.

**Results:**

A total of 10 RCTs were included, involving 1609 patients. The quality of the included studies was generally high. No significant difference was found between SILS and CLS in the postoperative complications, operation time, postoperative hospital stay, number of lymph nodes removed, readmission, reoperation, complication level I- II, complication level IIIa, complication level IIIb, prolonged Ileus, blood loss, infection, anastomotic leakage and operation time. The results showed that SILS group had a higher rate of intraoperative complications, but it had lower incision length and better cosmetic effects.

**Conclusion:**

These results indicate that SILS did not have a comprehensive and obvious advantage over the CLS. On the contrary, SILS has higher intraoperative complications, which may be related to the more difficulty of SILS operation, but SILS still has better cosmetic effects, which is in line with the concept of surgical development. Therefore, the SILS needs to be selected in patients with higher cosmetic requirements and performed by more experienced surgeons.

## Introduction

The incidence and mortality of colorectal cancer are disproportionately high, especially among men (1). In the past 60 years, general surgery has radically changed to minimally invasive surgery techniques to enhance the recovery rate, which became increasingly popular in the clinic (2). Conventional laparoscopy surgery (CLS) can decrease postoperative pain and accelerate patient recovery (3). Minimally invasive surgery has continued to play an important role as an alternative to traditional open surgery. Laparoscopic surgery demonstrated faster functional recovery rates, fewer postoperative complications, shorter length of the incision, and shorter hospital stay when compared with open surgery. Therefore, laparoscopic surgery has been recognized and recommended as a choice for colorectal cancer surgery without surgical contraindications (4). In order to pursue less trauma and better cosmetic effects, surgeons invented SILS in 2008. SILS not only strengthens the advantages of traditional laparoscopic surgery, but also has less surgical trauma, which represents the evolution of minimally invasive surgery towards scarless surgery (5, 6). However, Single-incision laparoscopy surgery (SILS) resented some new technical challenges compared with CLS (7, 8), for example, the limited number of working instruments which makes it difficult to achieve correct exposure and the necessary traction to tissues. Limited external working space, multiple instruments, and laparoscopies required for a procedure compete for the same space at the entry port, leading to external hand collisions and difficulty in internal manipulation of the instrument tip compared with CLS. Difficult to maintain pneumoperitoneum. The skills required for SILS differ from those required for CLS, SILS requires colorectal surgeons with superb laparoscopic skills, and surgeons need a learning curve cycle of 30-60 cases (9).

Whether SILS has advantages over conventional laparoscopy (CLS) is inconclusive. However several high-quality RCTs comparing single-incision with conventional laparoscopic surgery for colorectal cancer were reported. This systematic review and meta-analysis aimed to compare the efficacy and safety of SILS and CLS for colorectal cancer. The study included only RCTs.

## Methods

### Search strategy and inclusion criteria

We conducted this study according to the PRISMA guidelines (preferred reporting program for systematic review and meta-analysis), and obtained relevant information on SILS and CLS from PubMed, Embase, and Cochrane databases (10, 11). The following terms were searched: single port, single incision, reduce port, laparoscopy, laparoscopic surgery. In order to extend the search, the related-articles function was adopted. Only the most recent or complete report was adopted if multiple repeated studies were found. The search was completed on December 24, 2021. Comparative studies were included only if they had at least one available primary or secondary outcome for evaluation. Articles such as reviews, letters, editorials, case reports, animal experimental studies, and meeting abstracts were excluded.

First, all the identified titles and abstracts were examined by two independent reviewers. Next, the same two reviewers independently examined the full text of potentially relevant articles. In the event of disagreement, a third reviewer was consulted and the relevant articles were discussed until a consensus was reached.

### Data extraction and quality assessment

The following relevant information was extracted from all the included publications: reference, country/area, sample size, age, gender (M/F), BMI, tumor grade, study design. The main outcomes were intraoperative complications, postoperative complications. The secondary outcomes included operation time, postoperative hospital stay, number of lymph nodes removed, readmission, reoperation, complication level I-II, complication level IIIa, complication level IIIb, prolonged Ileus, blood loss, infection, anastomotic leakage, total incision length.

A 7-point Cochrane scale was used to assess the quality of the identified studies and includes seven assessment items: Random sequence generation (1 point), Allocation concealment (1 point), Blinding of participants and personnel (1 point), Blinding of outcome assessment (1 point), Incomplete outcome data (1 point), Selective reporting (1 point), Other bias (1 point), A score of 0 to 7 was assigned, and higher scores indicated higher quality. Any study scoring at least 4 was considered to have high methodology quality, and disagreements between the two researchers (Zeng DX and Li FH) were resolved by a third researcher (Tan L) who would make the final decision (**Tables 1**, **2**)

**Table 1:** Risk of bias summery.

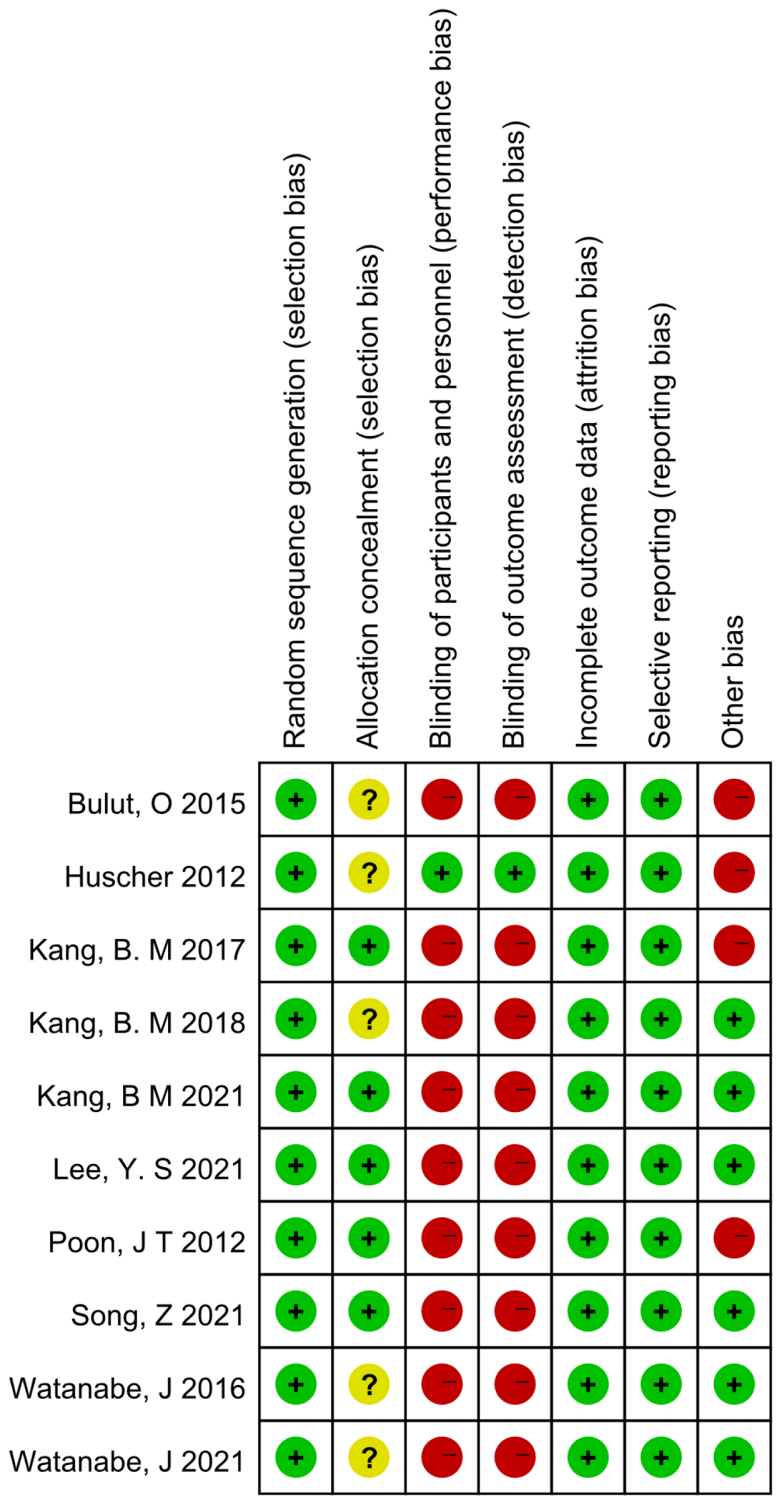

**Table 2:** Risk of bias graph.

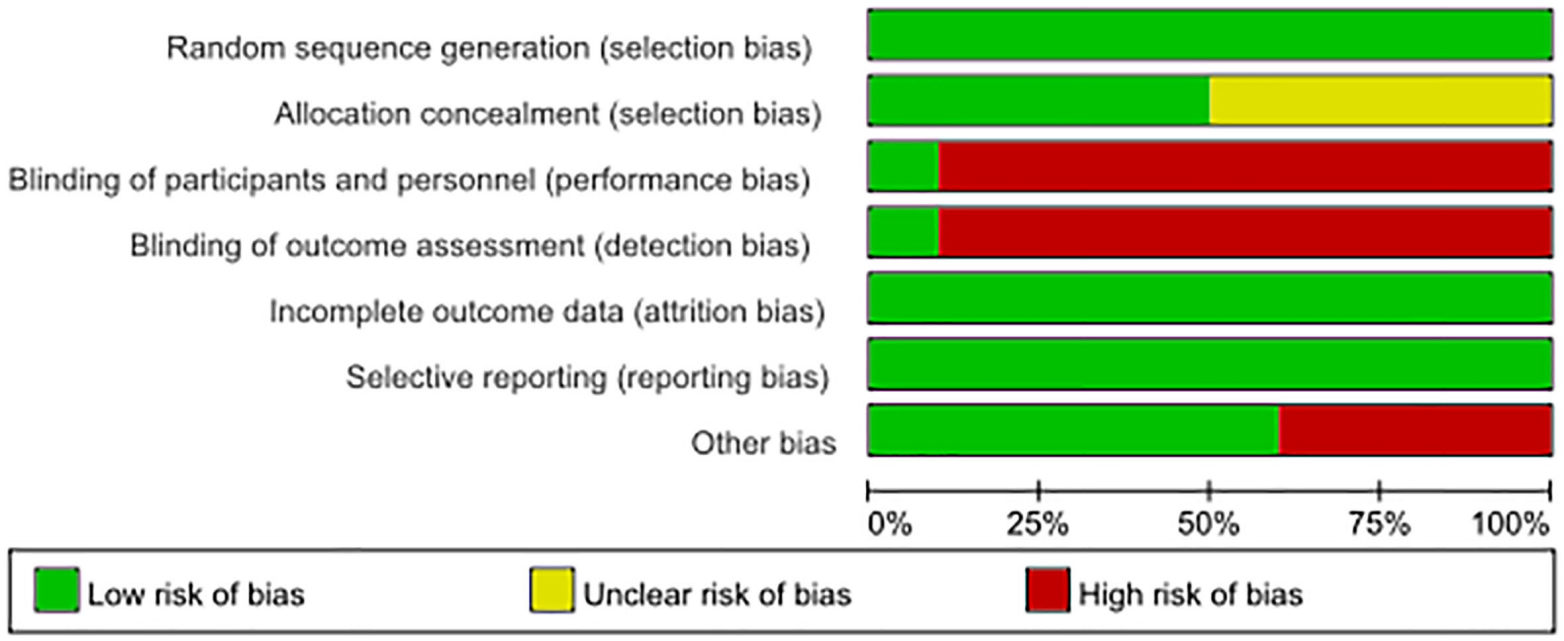

### Statistical analysis

Continuous variables and dichotomous variables were analyzed using weighted mean difference (WMD) and odds ratio (OR) respectively. The Chi^2^ and I^2^ statistics were used to assess the heterogeneity between studies, and the random-effects model was adopted if there was obvious heterogeneity between studies (p<0.05). Publication biases were determined using the funnel plot analyses. The analyses were performed with Review Manager (version 5.4.)

## Results

### Description of included and excluded studies

**Figure 1:**
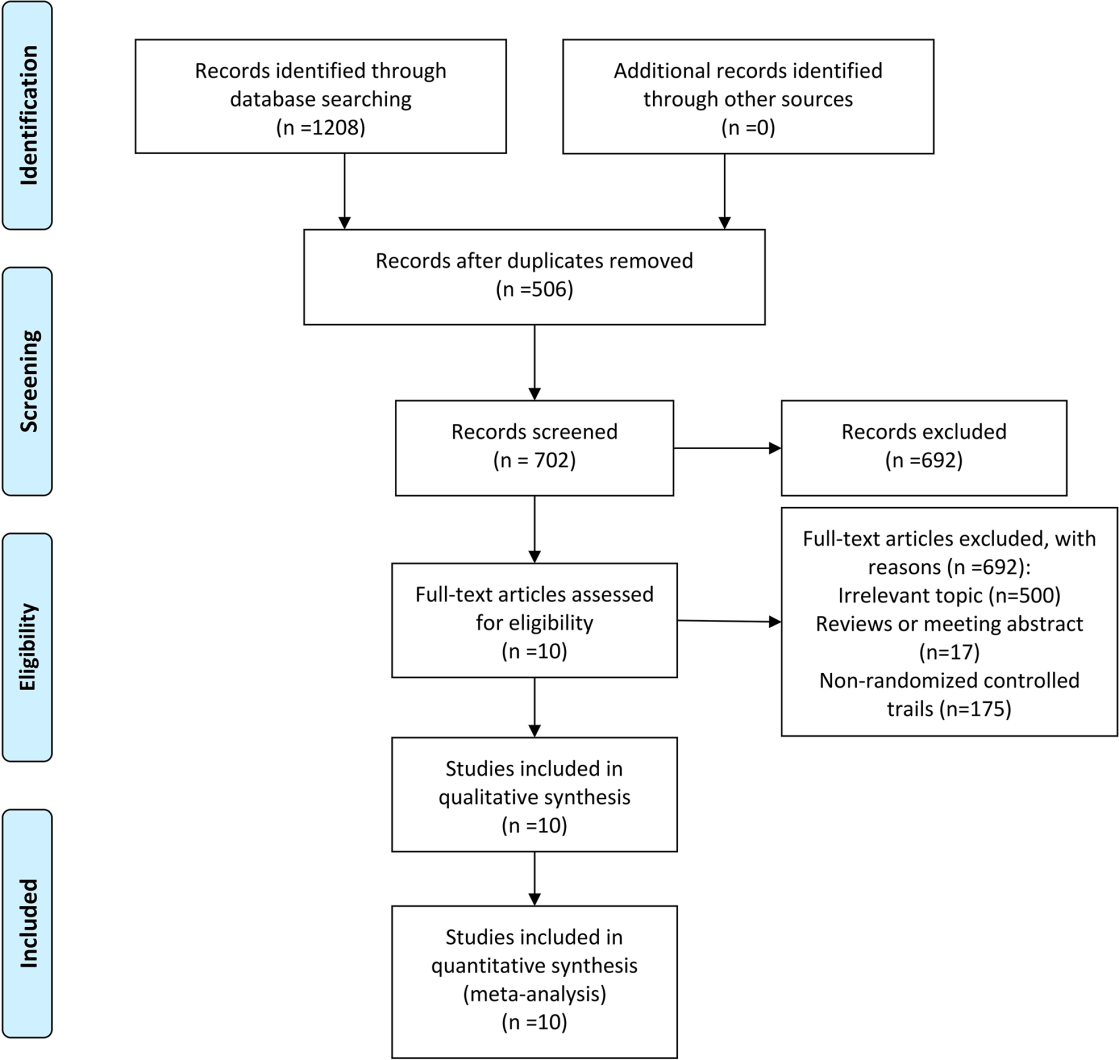
Inclusion and exclusion criteria chart.

Through the search strategy as shown in **Figure 1**, a total of 10 studies (12–21), including 1609 patients, were identified fulfilling the inclusion criteria. A total of 506 duplications were excluded at the stage of title and abstract review. Full texts of the remaining 702 studies were screened. Of these studies, 692 were excluded. These included 500 irrelevant topics, 17 reviews or meeting abstracts, and 175 non-randomized controlled trials.

### Patient demographics

Main characteristics of the selected studies included reference, country/area, sample size, age, gender (M/F), BMI, tumor grade, study design are as follows (**Table 3**).

**Table 3:** Main characteristics of the selected studies.

Country/Area	Sample size	Age	Gender (M/F)	BMI	Tumorgrade	Study design	Reference
Korea	359	63.4 (34–84) vs 62.6 (28– 85)	196/163	24.3 (17.0–32.0) vs 24.3 (18.0– 35.0)	Stage I-III	RCT	Lee, Y. S. 2021 (14), Kang, B. M. 2021 (15)
China	193	63 (54.5–69) vs 65 (56–70)	110/83	23.0 ± 2.8 vs 23.6 ± 3.2	Stage I-IV	RCT	Song, Z. 2021 (12)
Japan	200	66.6 ± 8.9 vs 66.7 ± 8.8	112/88	23.2 ± 3.3 vs 23.1 ± 3.3	Stage 0-III	RCT	Watanabe, J. 2021/2016 (13) (18),
Korea	181	62.4 (34–82) vs 62.3 (38– 85)	101/80	24.4 (17. 1–32.4) vs 24.2 (17.6– 34.1)	Stage I-III	RCT	Kang, B. M. 2018 (16)
Korea	62	63.2 ± 11.4 vs 62.2 ± 9.4	35/27	24.0 ± 3.0 vs 24.5 ± 3.0	Stage I-III	RCT	Kang, B. M. 2017 (17)
Denmark	40	69 (50–86) vs 73 (50–84)	16/24	24 (16–32) vs 24 (19–29)	StageI-III	RCT	Bulut, O. 2015 (19)
China	50	67 (37–83) vs 67 (57–81)	32/18	23.2 (16.9–28.8) vs 23.6 (16.5– 28.2)	Stage II-III	RCT	Poon, J T. 2012 (20)
Italy	32	70 ± 11 vs 70 ± 13	15/17	–	Stage I-III	RCT	Huscher. 2012 (21)

RCT, randomized controlled trial. BMI, body mass index. M/F, male/female.

## Meta-analysis results

### Primary outcomes

1. Intraoperative Complications

**Figure 2:**
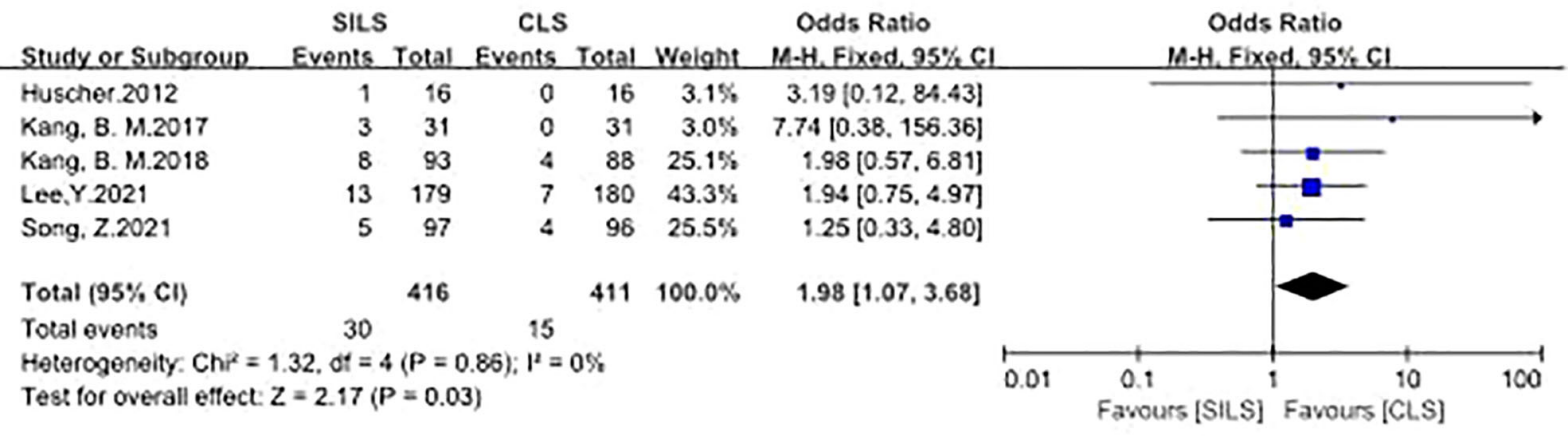
Meta-analysis of Intraoperative complications. SILS, single-incision laparoscopic surgery; CLS, conventional laparoscopic surgery.

Five studies participated the rate of intraoperative complications (12, 14, 16, 17, 21). Heterogeneity test: P = 0.86, I^2^ = 0%, showed no heterogeneity. The fixed-effect model was applied in the analyses. The results showed that the incidence of intraoperative complications in the SILS group was slightly higher than that in the CLS group. [OR = 1.98, 95% CI: 1.07 to 3.68, P = 0.03] (**Figure 2**).

2. Postoperative Complications

**Figure 3:**
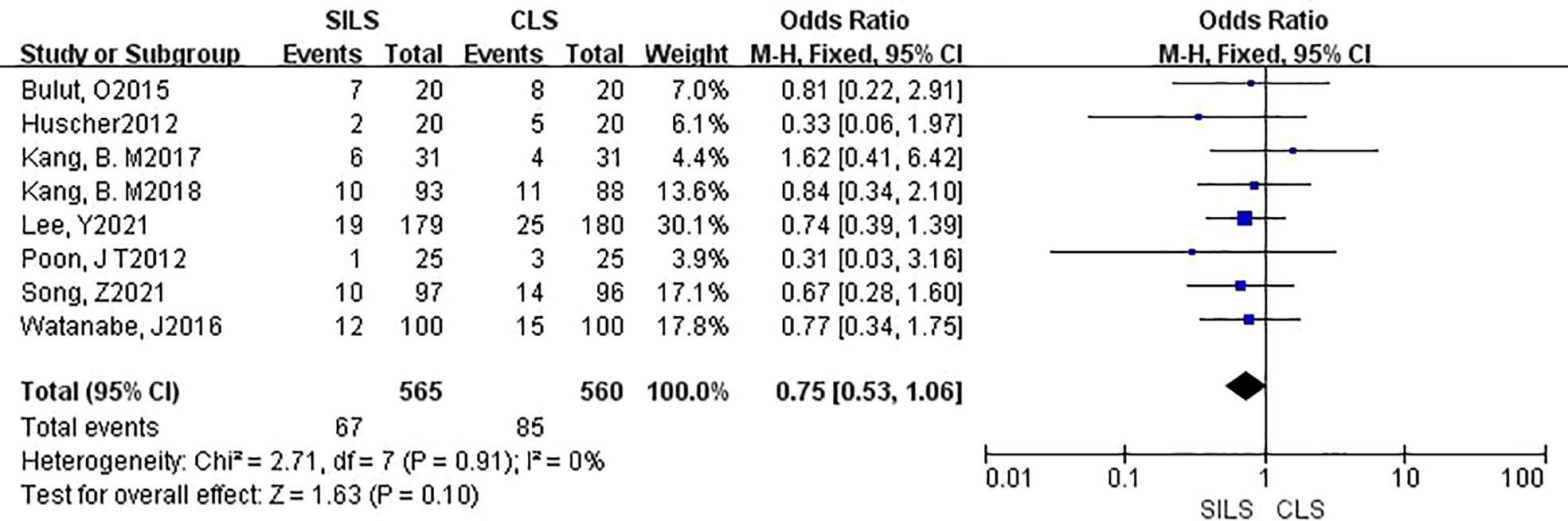
Meta-analysis of postoperative complications. SILS, single-incision laparoscopic surgery; CLS, conventional laparoscopic surgery.

A total of eight studies involving 1125 patients participated the rate of postoperative complications (12, 14, 16–21). Heterogeneity test: P = 0.91, I^2^ = 0%, showed no heterogeneity. Fixed-effect model was applied in the analyses. The results of Meta-analysis showed that there was no significant difference in postoperative complications between the SILS group and the CLS group [OR = 0.75, 95% CI: 0.34 to 1.06, P = 0. 10] (**Figure 3**)

### Secondary outcomes

3. Total Incision Length

**Figure 4:**
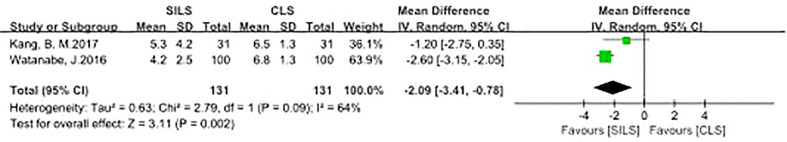
Meta-analysis of total incision length. SILS, single-incision laparoscopic surgery; CLS, conventional laparoscopic surgery.

Two studies with a total of 262 patients participated the rate of total incision length (13, 17, 18). Heterogeneity test: P = 0.09, I^2^ = 64%, showed significant heterogeneity. The random model was applied in the analyses. Compared with the CLS group, the SILS group had a shorter total incision length and better cosmetic effects. [MD = -2.09, 95% CI: -3.41 to -0.78, P = 0.002] (**Figure 4**).

4. Infection

**Figure 5:**
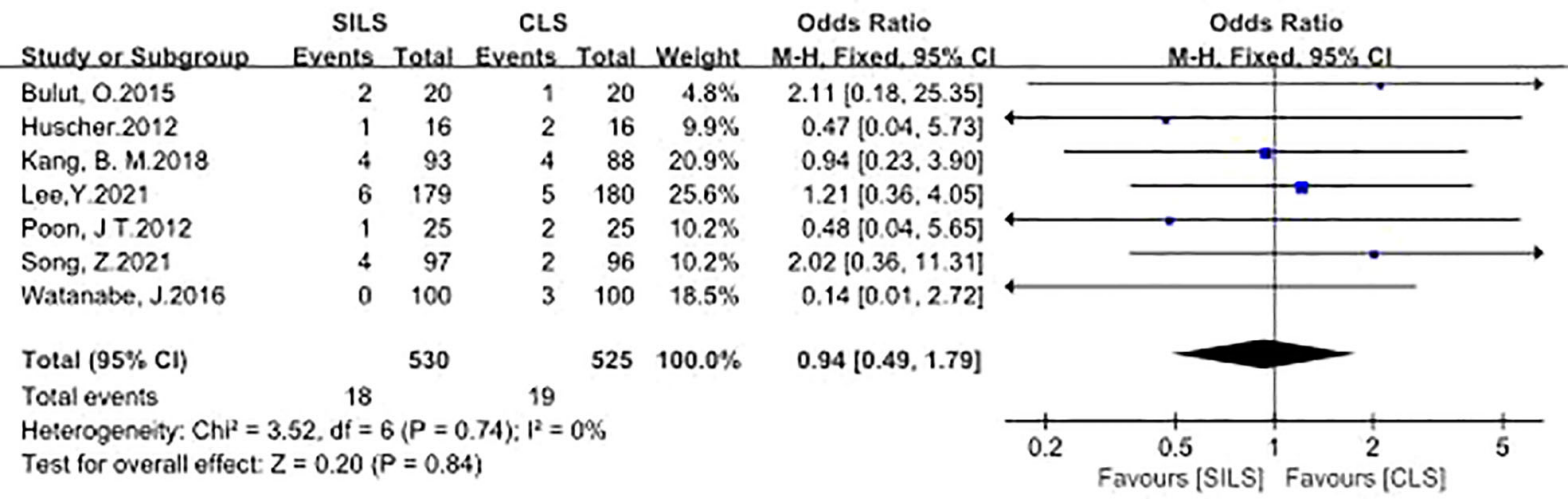
Meta-analysis of Infection. SILS, single-incision laparoscopic surgery, CLS; conventional laparoscopic surgery.

A total of seven studies with 1055 patients reported the rate of infection (12, 14, 16, 18–21). The details contained wound infection (12, 14, 16, 18–21), central venous catheters infection (12), bronchopneumonia (21), Heterogeneity test: P = 0.74, I^2^ = 0%, showed no heterogeneity. Fixed-effect model was applied in the analyses. The results of Meta-analysis showed that there was no significant difference in postoperative complications between the SILS group and the CLS group [OR = 0.94, 95% CI: 0.49 to 1.79, P = 0.84] (**Figure 5**).

5. Anastomotic Leakage

**Figure 6:**
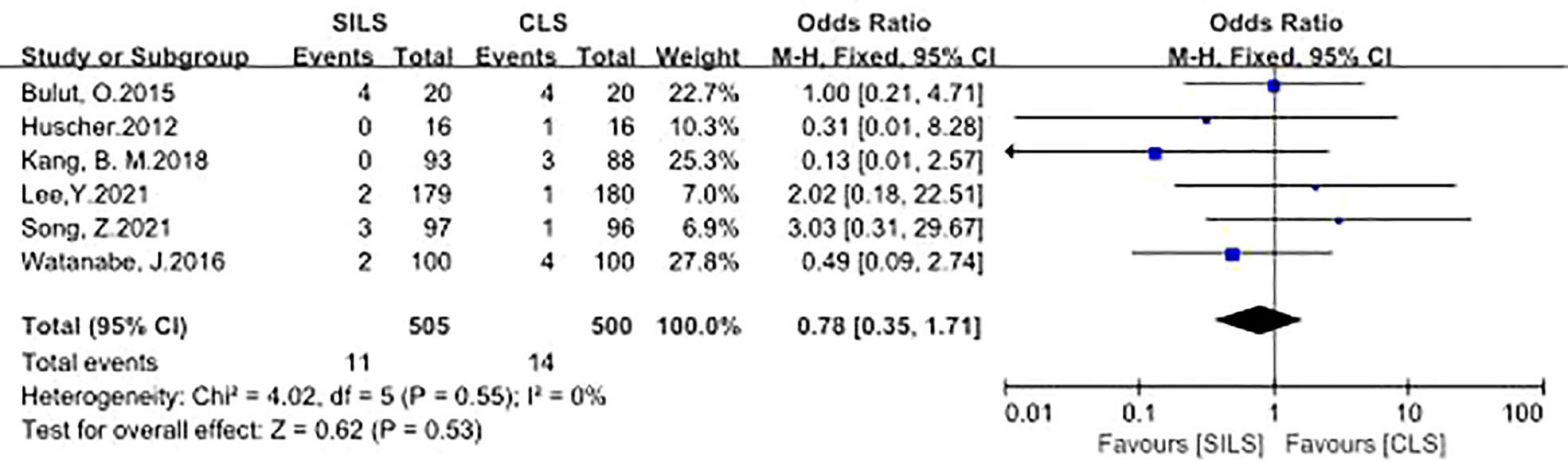
Meta-analysis of anastomotic leakage. SILS, single-incision laparoscopic surgery; CLS, conventional laparoscopic surgery.

A total of six studies with 1005 patients reported the rate of anastomotic leakage (12, 14, 16, 18, 19, 21). Heterogeneity test: P = 0.55, I^2^ = 0%, showed no heterogeneity. Fixed-effect model was applied in the analyses. Meta-analysis result showed that there was no significant difference between the two groups in the complications of anastomotic leakage [OR = 0.78, 95% CI: 0.35 to 1.71, P = 0.53] (**Figure 6**).

6. Operation Time

**Figure 7:**
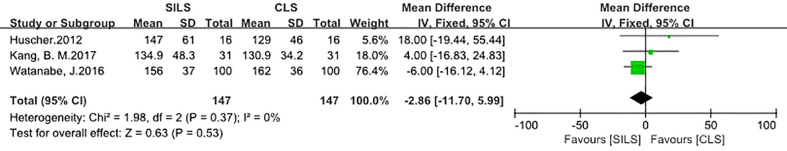
Meta-analysis of operation time. SILS, single-incision laparoscopic surgery; CLS, conventional laparoscopic surgery.

A total of three studies with 294 patients reported the rate of operation time (17, 18, 21). Heterogeneity test: P = 0.37, I^2^ = 0%, showed no heterogeneity. A fixed-effect model was applied in the analyses. Meta-analysis result showed that there was no statistical difference in operation time between the two groups [MD = -2.86, 95% CI: -11.70 to 5.99, P = 0.53] (**Figure 7**).

**Figure 8:**
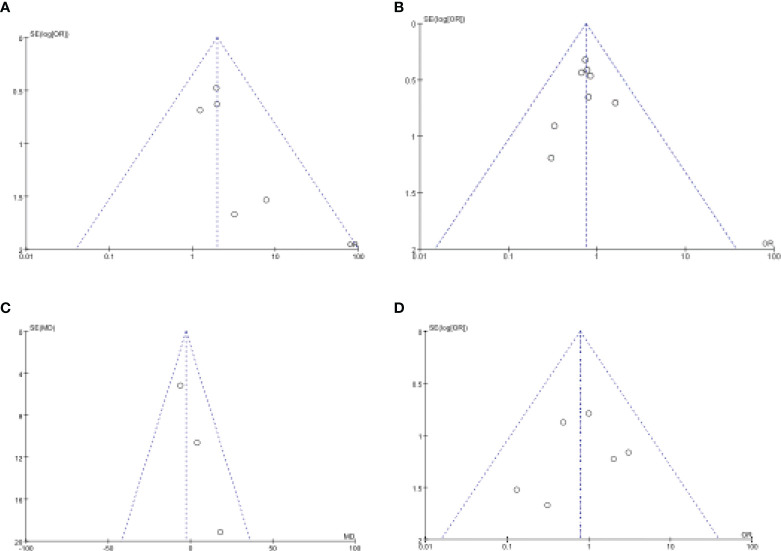
Funnel plot of publication bias in the meta-analysis. **(A)** Intraoperative Complications. **(B)** Postoperative Complications. **(C)** Operation Time. **(D)** Anastomotic Leakage.

**Figure 9:**
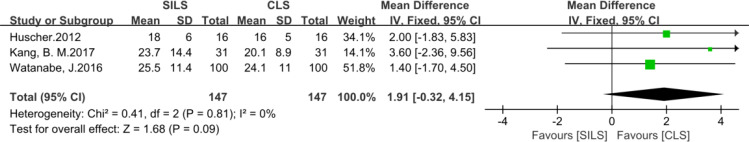
Meta-analysis of number of lymph nodes removed. SILS, single-incision laparoscopic surgery; CLS, conventional laparoscopic surgery.

**Figure 10:**
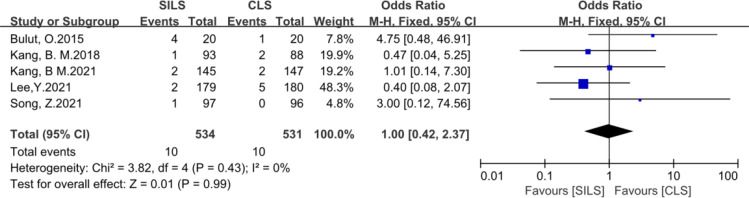
Meta-analysis of Readmission. SILS, single-incision laparoscopic surgery; CLS, conventional laparoscopic surgery.

**Figure 11:**
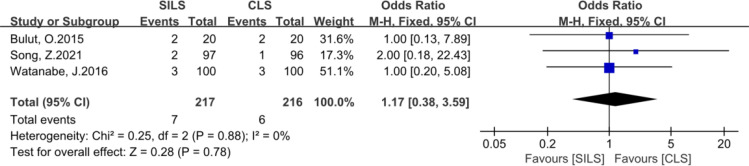
Meta-analysis of reoperation. SILS, single-incision laparoscopic surgery; CLS, conventional laparoscopic surgery.

**Figure 12:**
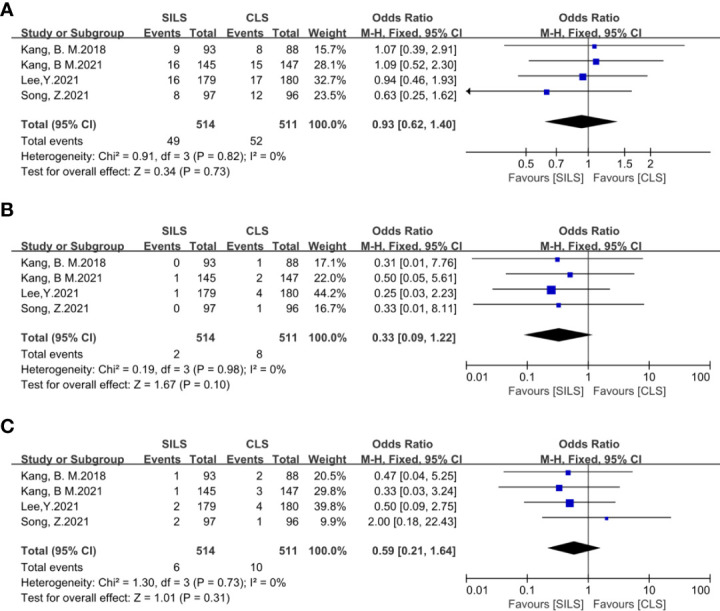
Meta-analysis of complication level I-III (**A**: I-II, **B**: IIIa, **C**: IIIb). SILS, single-incision laparoscopic surgery; CLS, conventional laparoscopic surgery.

**Figure 13:**
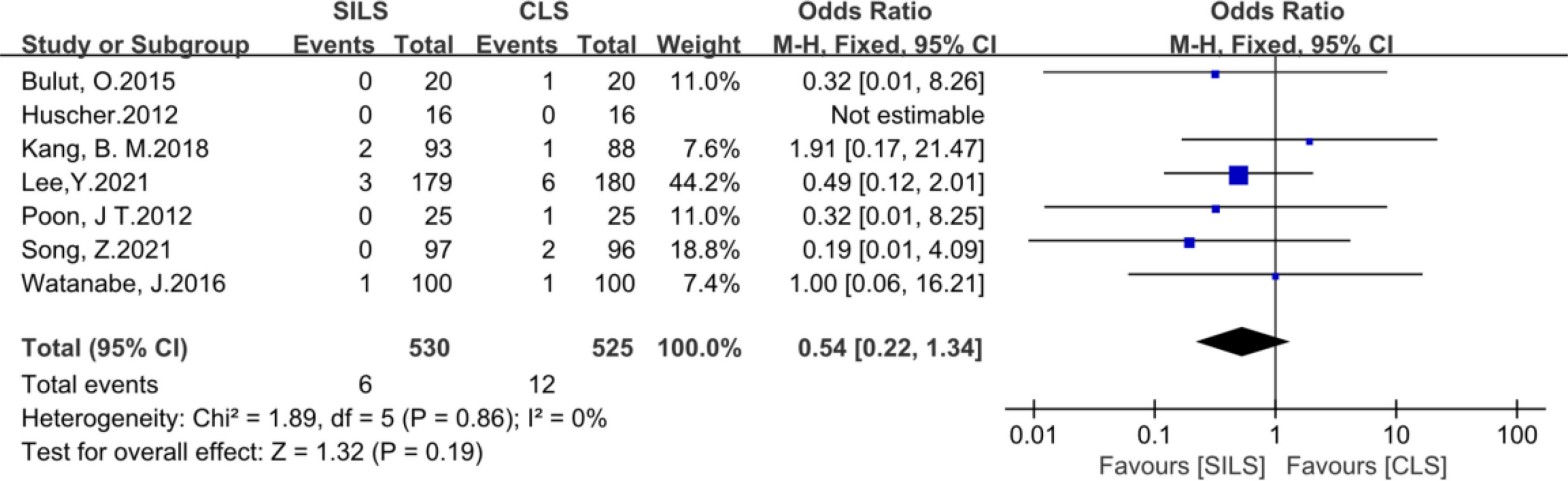
Meta-analysis of prolonged Ileus. SILS, single-incision laparoscopic surgery; CLS, conventional laparoscopic surgery.

7. The following metrics included postoperative hospital stay, number of lymph nodes removed, readmission, reoperation, complication level (I-III), Prolonged Ileus showed no statistical difference (**Figures 8**–**13**).

### Sensitivity analysis

Sensitivity analysis showed that there was no heterogeneity in remaining results except the total incision length (Heterogeneity test: P = 0.09, I^2^ = 64%). In our included articles, only two of them contained effective continuous variables, heterogeneity could not be analyzed by excluding any article.

### Publication bias

We evaluated publication bias by looking at the symmetry of funnel plots. Our results showed no publication bias (**Figure 8**).

## Discussion

Single-incision laparoscopic surgery (SILS) is an emerging minimally invasive technique (22). Patients and surgeons pay a lot of attention to it, because of its potential advantages such as smaller incision length, lower rate of postoperative complications, and so on (23, 24). However, SILS also has the same weaknesses as laparoscopic surgery, such as less tactile sense and limited instrument movement (25). These weaknesses are even enhanced during single incision laparoscopic surgery. The poor ergonomics and resulting technical difficulty with SILS contribute to the most important reason that this technique has not been rapidly adopted. When we talk about CLS, we are likely to consider that, comparing with SILS, it probably leads to a longer operative time, more intraoperative blood loss and higher intraoperative or postoperative complication rates. To determine whether SILS has advantages over CLS and whether its safety and efficacy are not inferior to CLS, we performed this meta-analysis of RCTs only.

In this study, we found that SILS had higher rates of intraoperative complications (vascular injury and adjacent organ injury). Besides the potential selection bias, on the one hand, this may be attributed to inadequate exposure of the surgical field and more difficult operation for surgeons. SILS combines multiple puncture holes of laparoscopy into a single hole, which violates the triangular operation control principle of laparoscopic technology. Single-incision operation requires colorectal surgeons to have superb laparoscopic skills. Usually, surgeons need a learning curve cycle of 30 to 60 cases to be proficient in application. Therefore, compared with CLS, SILS is more likely to lead to intraoperative complications, especially in some obese or difficult exposure cases. As for the obese patients especially BMI>30kg/m^2^, many studies agree that it’s a challenge for minimally invasive surgery, because abdominal exposure is poor compared with non-obese patients. However, the BMI of our cohort was relatively low, the patients with BMI>30kg/m^2^ was not enough to carry out statistical analysis.

On the other hand, complete mesocolic excision with central vascular ligation is essential for oncologic resection of right colon cancer (26), which needs to carefully clean the Henle superior mesenteric vein and artery and gastrocolic trunk. Anatomic variations of the blood vessels increases the challenges of operation as well. In addition, some previous studies reported that SILS is lack of triangular dissection, restricted degrees of freedom of movement the number of ports that can be used is restricted, the proximity of the instruments to each other during the operation-crossing fighting (16, 17, 21, 27), above factors may be more likely to lead to vascular injury in the SILS group. Single-port right hemicolectomy may be particularly challenging for SILS. There are certain technical differences between single-port right hemicolectomy and left hemicolectomy. Technical difficulty of the right hemicolon is more difficult to master, and the learning curve is slightly longer. As for the subgroup analysis of SILS, Lee,Y et al. reported that right hemicolectomy was associated with more operative complications than anterior resection with SILS (14). Because Complete mesocolic excision with central vascular ligation is vital of importance, which carefully dissection around the superior mesenteric vein and artery and gastrocolic trunk of Henle is required. In addition, The above vessels are prone to anatomical variation. These undoubtedly increase the difficulty of laparoscopic surgery, especially magnified in SILS because of lack of triangular dissection and difficulty achieving traction and countertraction in the SILS group. Therefore, to address a potential technical bias regarding learning curve, all procedures were performed by expert senior surgeons with good experience of both SPL and MPL techniques (28). However, Say-June Kim et al. showed lower rates of intraoperative complications, they agreed that a significant disadvantage of conventional laparoscopic surgery is the separation of the operator’s hands and eyes. Conventional laparoscopic surgery provides a surgical area controlled by a human assistant. This easily leads to unsatisfactory interactions between the operation and the assistant surgeon, which can compromise optimal visual field (29). On the contrary, it’s beneficial to SILS especially for skilled surgeons. Of course, this also requires surgeons to spend more time training to master the application of this technology. Khayat, A et al. were cautious about the two groups of similar results of their own experiments, because of selection bias, the patients operated with the SILS approach might be easier cases (30). This may compensate for deficiencies of SILS in other areas, resulting in equal complications in both groups.

This meta-analysis showed that SILS had less total length of skin incision than CLS [MD = -2.09cm, P = 0.002]. The umbilical cord is a natural scar, keeping the incision in the umbilical ring could improve the cosmetic effect. SILS not only strengthens the advantages of traditional laparoscopic surgery, but also has less surgical trauma, representing the evolution of minimally invasive surgery towards scarless surgery. This is also the advantage and value of this technology. However, Bush et al’s study (31) investigating women’s satisfaction for minimally invasive surgery (conventional vs. single-incision vs. robotic surgery) showed that the proportion of the CLS was the highest (56.4%) and the second was SILS (41. 1%). The comparative assessment of long-term complications (SILS-related incisional hernia) was not addressed in our study, but a recent RCT study suggested no significant difference in the incidence of incisional hernia after SILS arm versus SILS arm versus CLS arm with a long-term follow-up (13). Whether SILS has advantages in the following aspects needs further high-quality evidence: such as Less post operative pain, Less wound related complications, Faster recovery, Early return to work. The continuous data in the most of the introduced studies are in the form of median and extreme values which can’t be imported into the forest map, thus the conclusion is one-sided to some extent. Further research and better evaluation methods are needed to confirm the results.

In the present study, it showed a similar operation time for the SILS group to the CLS group [MD: -6min] which did not differ significantly (p=0.53), Some previous studies are similar to ours (17, 18, 32), However, some research required a longer time (33–35) while others required a shorter time than the CLS group (18, 36), besides the potential selection bias, The reasons for the rich results may be as follows, On the one hand, surgeons who focus on SILS and manage more surgical cases will reach a higher level of expertise, and the accumulating experience may considerably decrease operative time (37, 38), on the contrary, inexperienced surgeons may need more time. Yimei Jiang et al. reported that their surgeon was skilled in 3-port laparoscopic colorectal surgery before performing SILS for colorectal cancer, which is helpful for overcoming the learning curve of SILS. On the other hand, because of the reduced length and number of apertures of the skin incision, it takes less time for both making and closing the skin incision in SILS. All in all, though SILS for rectal cancer is much more difficult than CLS, surgeons accumulated more surgeries, overcame the learning curve of single-incision laparoscopy, a further reduction in operating time is only a matter of time.

This meta-analysis did not confirmed that SILS reduced the rate of postoperative complications. However, some studies showed different results, Bulut et al. pointed out opposite results. We analyzed the factors leading to this result from many aspects. First, there is no uniform standard for patient inclusion. Postoperative complications such as bleeding, incision infection and anastomotic fistula were related to individual inclusion criteria such as age, nutritional status, underlying diseases and tumor scope. Under the same operation, the postoperative complications of good individual condition were significantly less than those of poor individual condition, so the occurrence of postoperative complications was difficult to be measured by the difference of surgical methods (9). Second, although SILS has a shorter incision length and fewer incisions than CLS (39), the improvement of equipment in recent years has led to more and more changes in the skills of surgeons. Shorten the operation time, shorten the length of the incision, reduce postoperative pain, promote early activity to enhance recovery. All these can reduce the incidence of postoperative complications. Third, patients with different postoperative nursing specifications, postoperative hospital and family nursing support will affect the occurrence of postoperative complications. Kang,B,M et al. have published two RCTs with different conclusions on postoperative complications, so we believe that the occurrence of postoperative complications is not related to the effect of operation type.

In this meta-analysis, only 2 studies provided data on length of hospital stay. Because the included studies did not use the same discharge criteria and had a low sample size, differences in length of stay were of low reference value (9). In condition, different surgical instrument specifications and inconsistent surgical methods for colorectal cancer (such as low rectal cancer anterior resection, radical abdominal perineotomy combined with rectal cancer) may also affect the operative time and incidence of intraoperative complications. All of these indirectly increase the length of hospital stay. Therefore, it is difficult to accurately evaluate the advantage of SILS in hospital stay.

Due to insufficient data from included randomized controlled studies, the long-term outcomes of SILS was not evaluated. Some previous studies showed that the long-term outcomes of patients undergoing SILS were comparable to those of patients undergoing CLS (40–42). Hirano, Y. et al. reported the median follow-up interval was 60 months. The 5-year relapse free survival for stage I, stage II and stage III disease were 90.5%, 88.1% and 79.6%, respectively. The 5-year overall survival for stage I, stage II, stage III and stage IV disease were 97.6%, 92.9%, 88.6% and 40.9%. The factors of long-term outcomes are comprehensive. Besides the operation factors, postoperative care and personalized chemo-radiotherapy are essential as well. More randomized controlled trials are needed to demonstrate the advantages and disadvantages of long-term outcomes in patients with SILS.

Compared with other previous meta-analysis (43–45) including retrospective studies or clinical controlled trials (CCTs), this meta-analysis only included and analyzed all relevant RCTs in the present to ensure that the results were more reliable. However, this study has some limitations. First, our searched database is limited, some open data is not included, and included articles had few data samples. Second, each surgeon had a different technical proficiency which might be likely to cause the data of the article to be offset. Thirdly, only one study blinded the participants, while the others were open-labeled RCTs, which can lead to substantial implementation bias. What’s more, the included literature lacks long-term follow-up results, including the rate of local tumor recurrence or distant metastasis, and survival rate.manipulation of the instrument tip compared with CLS.

## Conclusion

SILS showed shorter incision length and higher rates of intraoperative complications compared with CLS, it means that SILS has good cosmetic effects with relatively high surgical risk. Therefore, SILS needs to be selected in patients with higher cosmetic requirements and performed by more experienced surgeons.What’s more, systematic review and meta-analysis did not prove that the SILS had a comprehensive and obvious advantage over the CLS. Some extra metrics such as postoperative patient recovery, postoperative hospital stay, postoperative pain may have potential benefits which needs to be further explored by high-quality RCTs.

## Data availability statement

The original contributions presented in the study are included in the article/supplementary material. Further inquiries can be directed to the corresponding authors.

## Author contributions

Author contributions: F-hL and D-xZ performed data acquisition, analysis, and interpretation and drafted the manuscript; LC contributed to data interpretation and revised the manuscript; XC-f Provided suggestions for modification; TL and ZP aimed to revise the article; J-wX contributed to the study conception and design and critical revision of the manuscript; all authors approved the final version of the submitted manuscript.

## Funding

National Natural Science Foundation of China, NO. 81070378 and NO. 81270561; Special Research Fund for the First Affiliated Hospital of Chengdu Medical College, NO. CYFY2019YB08; and High-level Talents Introduction Fund for the First Affiliated Hospital of Chengdu Medical College, NO. CYFY2018GQ17.

## Conflict of interest

The authors declare that the research was conducted in the absence of any commercial or financial relationships that could be construed as a potential conflict of interest.

## Publisher’s note

All claims expressed in this article are solely those of the authors and do not necessarily represent those of their affiliated organizations, or those of the publisher, the editors and the reviewers. Any product that may be evaluated in this article, or claim that may be made by its manufacturer, is not guaranteed or endorsed by the publisher.
